# Concept and implementation of the longitudinal mosaic curriculum planetary health at the Faculty of Medicine in Würzburg, Germany

**DOI:** 10.3205/zma001615

**Published:** 2023-05-15

**Authors:** Jörg Schmid, Anna Mumm, Sarah König, Janina Zirkel, Eva-Maria Schwienhorst-Stich

**Affiliations:** 1 University Hospital Würzburg, Department of General Practice, Würzburg, Germany; 2 University of Ulm, Ulm, Germany; 3 University Hospital Würzburg, Institute of Medical Teaching and Medical Education Research, Würzburg, Germany; 4 University of Würzburg, Teaching Clinic of the Faculty of Medicine & University Hospital Würzburg, Institute of Medical Teaching and Medical Education Research, Würzburg, Germany

**Keywords:** planetary health, planetary health education, medical education, curriculum development, climate change and health

## Abstract

**Background of the project:**

Stakeholders in healthcare and science increasingly demand the rapid integration of teaching content on planetary health (PIH) into the curricula of all the healthcare professions. In medical education, such topics are currently only covered inadequately and are mostly limited to elective courses.

**Why was the project initiated?:**

In order to reach all medical students in the sense of a learning spiral and promote an interdisciplinary understanding of planetary health, a longitudinal mosaic curriculum is being developed that introduces aspects of planetary health throughout the entire course of study. We share the first experiences of the start of this project as an example to inspire similar activities elsewhere.

**Implementation of the project:**

We mapped all the courses at the Faculty of Medicine in Würzburg and compared them with existing learning objectives on planetary health topics from the National Competency-Based Catalog of Learning Objectives for Medical Education. We then identified curricular injection points and held consultations with teaching staff and course coordinators from 26 different specialities in order to integrate the respective contents into the courses and, if necessary, develop new content. An overview of all curricular injection points with the corresponding topics, learning objectives, and teaching and examination methods is under development.

**Evaluation of the project:**

The lecturers exchanged ideas with the project team of the teaching clinic of the Faculty of Medicine; further networking meetings to coordinate a learning spiral are to follow. The lecturers were asked to provide structured learning objectives in the categories “knowledge”, “attitudes”, “skills”, and “confidence” on the topics integrated into the courses. Oral as well as written evaluations using Evasys^®^ questionnaires among students and lecturers are planned.

**Final overall assessment, outlook:**

Planetary Health topics have been introduced in several courses following our intervention. In the context of a learning spiral, teaching staff from further medical disciplines will be contacted so that more perspectives can be highlighted at different points in the curriculum. In addition, interdisciplinary teaching formats will be developed in order to take the complexity of the interrelationships into account.

## Zusammenfassung

**Was ist der Kontext bzw. Hintergrund des Projekts?:**

Die rasche Integration von Lehrinhalten zu Planetary Health (PlH) in die Curricula aller Gesundheitsberufe wird zunehmend von Stimmen aus Gesundheitsberufen und Wissenschaft gefordert. In der Lehre der Humanmedizin sind diese Themen bisher unzureichend und bislang meist nur in Wahl(pflicht)fächern abgebildet.

**Warum wurde das Projekt initiiert?:**

Um alle Medizinstudierenden im Sinne einer Lernspirale zu erreichen und ein interdisziplinäres Verständnis von Planetary Health aufzubauen, soll ein longitudinales Mosaik-Curriculum etabliert werden, das Aspekte von Planetary Health im gesamten Studienverlauf verortet. Wir teilen in diesem Artikel erste Erfahrungen aus der Anfangsphase des Projekts, um zu ähnlichen Vorhaben andernorts zu inspirieren.

**Wie wurde das Projekt umgesetzt?:**

Es erfolgte ein Mapping aller Lehrveranstaltungen an der Medizinischen Fakultät Würzburg. Diese wurden mit bestehenden Lernzielen zu Themen der planetaren Gesundheit im Nationalen Kompetenzbasierten Lernzielkatalog Medizin 2.0 abgeglichen und curriculare Verortungspunkte identifiziert. Daraufhin wurden mit bereits 26 Lehrverantwortlichen Gespräche geführt, um die jeweiligen Inhalte in die Lehrveranstaltungen zu integrieren sowie ggf. neue zu entwickeln. In einer Übersicht sollen alle curricularen Verortungspunkte mit dazugehörigen Themen, Lernzielen sowie Lehr- und Prüfungsmethoden zusammengefasst werden.

**Wie wurde das Projekt evaluiert?:**

Die Dozierenden tauschten sich mit dem Projektteam der Lehrklinik an der Medizinischen Fakultät aus, weitere Vernetzungstreffen zur Abstimmung einer Lernspirale sollen folgen. Von den Dozierenden wurden strukturiert Lernziele in den Kategorien „Wissen“, „Haltungen“, „Fertigkeiten“ und „Selbstvertrauen/Selbstwirksamkeit (Confidence)“ zu den in die Lehrveranstaltungen integrierten Themen abgefragt. Mündliche sowie schriftliche Evaluationen mittels Evasys^®^-Fragebögen unter Studierenden und Dozierenden sind geplant.

**Abschließende Gesamteinschätzung, Ausblick:**

Gemeinsam mit den Dozierenden konnten bereits in mehreren Lehrveranstaltungen Themen planetarer Gesundheit eingebracht werden. Im Sinne einer Lernspirale sollen Lehrverantwortliche weiterer Fächer kontaktiert werden, sodass verschiedene Blickwinkel an unterschiedlichen Stellen im Curriculum beleuchtet werden. Außerdem sollen interdisziplinäre Lehrformate entwickelt werden, um der Komplexität der Zusammenhänge Rechnung zu tragen.

## 1. Background of the project

Global environmental changes such as global warming and biodiversity loss pose a massive and unprecedented threat to human health in the twenty-first century [1]. For example, mortality increases during more frequent heat waves, there is a rise in certain vector-borne infectious diseases, and increasingly frequent extreme weather events can lead to physical and psychological trauma. To date, these interrelationships are inadequately represented in medical school curricula. Therefore, the policy brief of the Lancet countdown [2], as well as the German Medical Association [3], among others, call for a rapid implementation of planetary health education in the curricula of all health professions. Planetary health refers to “the health of human civilization and the natural systems on which it depends” [4]. Exam questions on planetary health can be expected to increase in state and faculty examinations in the future, now that the new National Competency-Based Learning Objective Catalogue of Medicine (NKLM 2.0) ([https://www.nklm.de], retrieved at 30.07.2022), includes numerous aspects on planetary health in the application examples ([https://www.med.uni-wuerzburg.de/planetaregesundheit/aktivitaeten/longitudinales-curriculum-planetare-gesundheit-an-der-medizinischen-fakultaet/], retrieved at 30.07.2022). The Institute for Medical and Pharmaceutical Examination Questions (IMPP) established the interprofessional working group on climate, environment, and health in early 2021. The Amboss learning platform, which is widely used among medical students in Germany, has also published a detailed article on planetary health. So far, however, these aspects have been insufficiently represented in medical education and are offered mostly in elective courses, where they reach only a small proportion of students. 

## 2. Why was the project initiated?

Several medical faculties, for instance in Greifswald, Mannheim, Berlin, Ulm, or Tübingen, offer electives on planetary health, but these are mainly taken by students who are already interested. The students of the Würzburg elective *Planetary Health: Climate.Environment.Health*, which was introduced in 2021, have frequently requested for the topics to be taught in more detail in the core curriculum. Aspects of planetary health have explicitly been taught in lectures on environmental medicine in Würzburg since 2021. With a limited number of hours in a few lectures, however, it is not possible to develop a comprehensive understanding of the interdisciplinary relations. Many lecturers also often lack the conceptual understanding of the interrelationships of planetary health, expertise, or time resources to integrate the complex and new topics into their teaching. The literature that offers practical tips of how to best implement those is currently increasing [5], [6].

In order to reach all students in a curriculum and to build up a holistic understanding of the connections between global environmental change and human health, the Würzburg longitudinal mosaic curriculum planetary health is currently being developed. In the future, the individual aspects of planetary health are to be located in all semesters (longitudinally) and are to be integrated into different subjects (mosaic) in the context of a learning spiral.

## 3. Implementation of the project

A physician (JS) was employed from May to December 2021 at a 20% staff position, supported by a student assistant to implement the project.

In June and July 2021, a public four-part online event series was conducted as an introduction to planetary health for health professionals. All teaching staff at the Würzburg Medical Faculty and other persons at the Würzburg University Hospital were invited to participate.

In parallel, the Würzburg curriculum was reviewed in terms of

1. the existing application examples with reference to topics on planetary health of the NKLM 2.0, as well as
2. the catalog of national planetary health learning objectives of the NKLM [7].

This process involved local medical students and was coordinated with the local medical student council.

Our team had incorporated new lectures on planetary health (three units) in the course of environmental medicine starting in the summer semester 2021. In this context, we surveyed via online questionnaires (see attachment 1 ) whether the students were interested in integrating the topics into other curricular courses. The high interest of the students in curricular teaching on planetary health served both as a needs analysis and as a basis for further curricular development activities. Initial discussions took place with teaching supervisors in the following subjects (number of semester in parentheses): biology (1), medical psychology (2), biochemistry (2), physiology (3), toxicology (5), environmental medicine (5), microbiology (5), history and ethics (6), preventive medicine (6), internal medicine-endocrinology (6), internal medicine-gastroenterology (6), health economics (6), internal medicine-nutritional medicine (7), internal medicine-cardiology (7), internal medicine-pulmonology (7), internal medicine-nephrology (7), otorhinolaryngology (7), dermatology (7), anesthesiology (8), pediatrics (8), infectious diseases (8), gynecology (9), neurology (9), psychiatry (9), and geriatrics (9). Further consultations with other disciplines follow.

Based on the first German textbook planetary health [8], which was published in September 2021, and based on our own literature searches in the PubMed literature database, a concept paper with potential learning objectives and injection points was prepared for all identified curricular subjects. This was emailed to the identified teaching supervisors with an invitation to a meeting. In a video conference lasting approximately 60 minutes, the respective structural requirements, content focuses, and special circumstances of the individual subjects were then explored with the teaching supervisors, and support was offered for the creation of teaching materials. Next steps, potential learning objectives, and already established aspects were recorded in a meeting protocol.

The core of the project is a tabular overview in which all curricular injection points are summarized with associated topics, learning objectives, teaching, and examination methods. In a first step, the team concentrated on the rapid integration of teaching content into existing lectures or on the creation of new lectures. In a second step, courses for applied and communication skills related to planetary health are to be designed and integrated into the curriculum. This will be done in the context of the curricular restructuring based on the upcoming German licensing regulations (Approbationsordnung) in the next few years. Transdisciplinary and interprofessional courses have to be developed as well.

At the time of writing, definitive learning objectives are being formulated for all subjects in consultation with course supervisors and instructors. Based on the proposals of the AMEE consensus statement [9], these objectives will address the dimension of confidence in addition to the usual learning objective dimensions of knowledge, skills, and attitudes (see table 1 (Tab. 1)).

**Figure 1:**
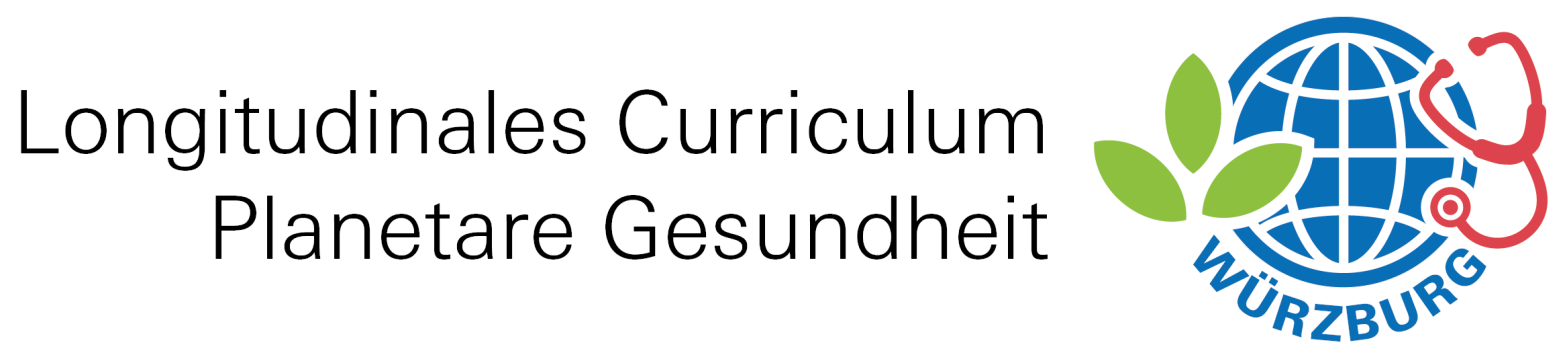
Logo of the Würzburg longitudinal mosaic curriculum planetary health that all lecturers can use for their teaching materials.

For students to make better connections between the topics and to establish transparency, a logo designed specifically for this purpose can be placed on the respective slides (see figure 1 (Fig. 1)).

## 4. Evaluation of the project

Exchange meetings of all contacted lecturers are planned for sharing of best-practice examples, clarification of questions and harmonization of content and teaching objectives across the curriculum. After the planned implementation of most of the planned innovations, the project team will provide evaluations to participating lecturers to systematically receive student feedback. These two sources of feedback will be used to improve and refine the curriculum in the long term. 

## 5. Final overall assessment and outlook

In the discussions with the lecturers, we have received much positive feedback and interest in the project so far. They explicitly appreciated that the Faculty of Medicine supports and coordinates this curricular expansion. Many consider it to be tremendously important.

The authors observe a local transformation process in Würzburg that consists of networking within the faculty and of the establishment of new research collaborations. The teaching project highlights the topic at the university and the university hospital, it is perceived as pioneering work. However, well-equipped staff resources are needed to coordinate the time-consuming process. Above all, integration into the upcoming curricular restructuring on the basis of the German licensing regulations (Approbationsordnung) must be coordinated.

There is a high demand for exchange of experiences in developing and implementing courses on planetary health in the medical core curriculum at other universities as well. This project outline can therefore contribute significantly by sharing our experience with other faculties. The current and anticipated curricular restructuring in light of the new medical licensing regulations provides an ideal window of opportunity to address the demands to integrate the important topics of planetary health into the medical curricula as soon as possible. 

## Acknowledgements

We thank Dr. med. Claudia Löffler and Prof. Dr. Stefan Frantz for contributing their newly developed teaching objectives to display as an example in table 1 (Tab. 1).

## Competing interests

The authors declare that they have no competing interests. 

## Supplementary Material

Questions from questionnaire in the summer semester of 2021 after the lecture “Planetary Health” in the Environmental Medicine lecture series (n=130 students in semester 5)

## Figures and Tables

**Table 1:**
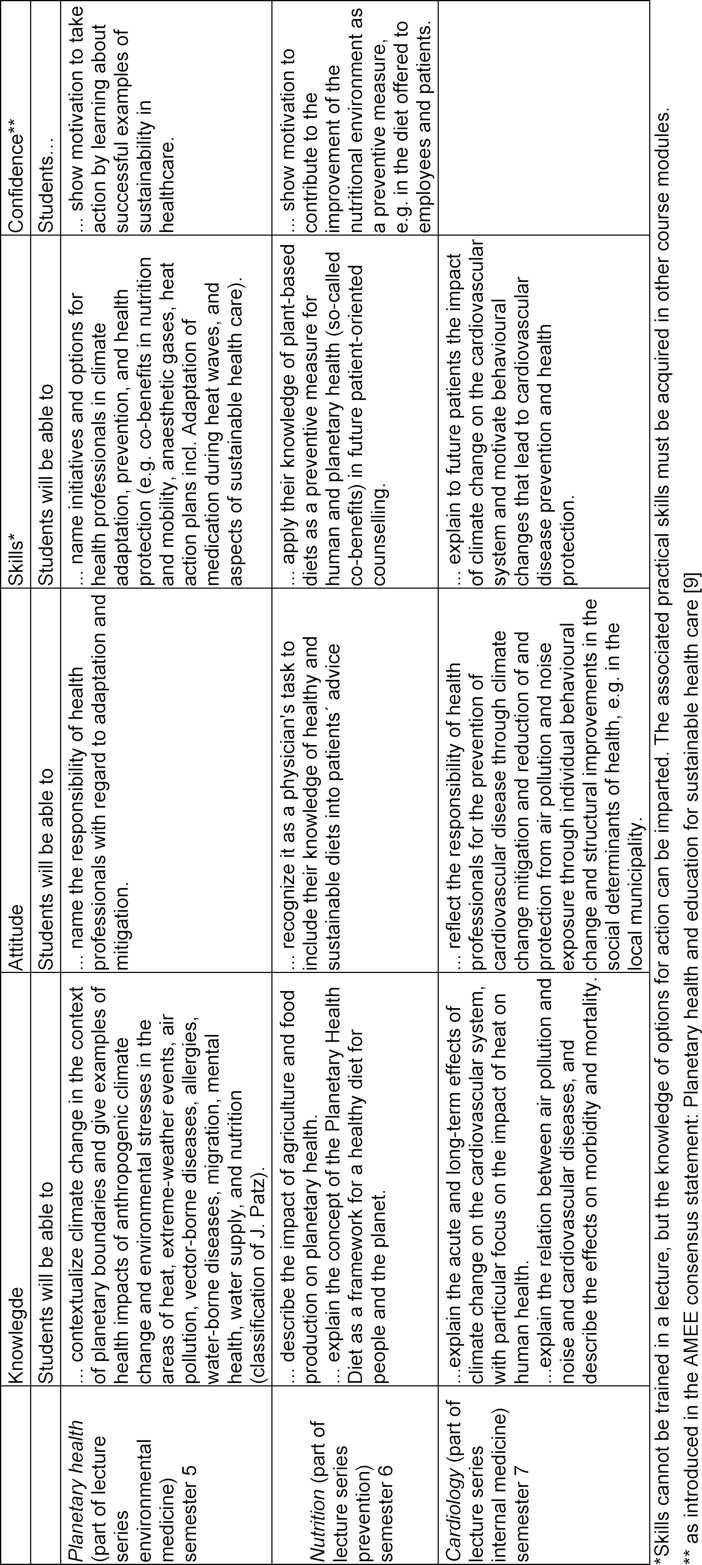
Examples of learning objectives for environmental medicine, prevention and cardiology
