# Supplementary material for: Concept and implementation of the longitudinal mosaic curriculum planetary health at the Faculty of Medicine in Würzburg, Germany
Source: GMS J Med Educ. 2023 May 15;40(3):Doc33. doi: 10.3205/zma001615 (PMC10291347; doi:10.3205/zma001615)
Supplement: Questions from questionnaire in the summer semester of 2021 after the lecture “Planetary Health” in the Environmental Medicine lecture series (n=130 students in semester 5) [file JME-40-33-s-001.pdf]

Attachment 1: Questions from questionnaire in the summer semester of 2021 after the lecture "Planetary Health" in the Environmental Medicine lecture series (n=130 students in semester 5)

Translation:

Question 1: I think the content of the lecture is relevant for medical education:

compelety disagree - - - - - completely agree

Question 2: The content is adequately integrated into the medical studies:

compelety disagree - - - - - completely agree

Results presented

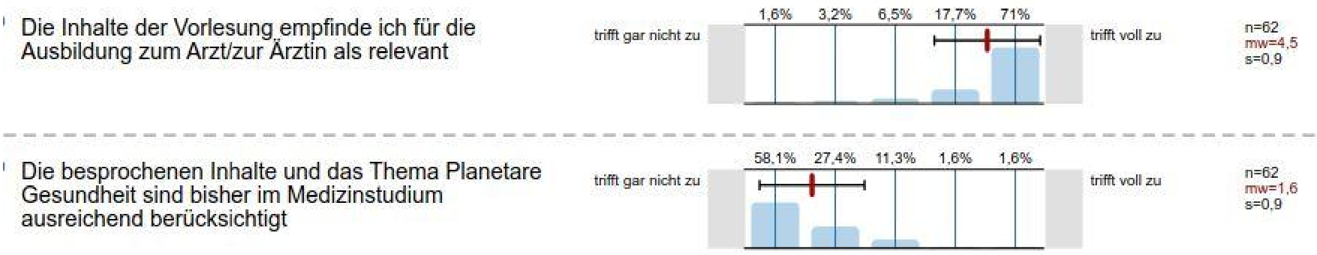

Attachment 1 to Schmid J, Mumm A, König S, Zirkel J, Schwienhorst-Stich EM. *Concept and implementation oft he longitudinal mosaic curriculum planetary health at the Faculty of Medicine in Würzburg, Germany.* GMS J Med Educ. 2023;40(3):Doc33. DOI: 10.3205/zma001615
